# Supplementary material for: Structural domain in the Titin N2B-us region binds to FHL2 in a force-activation dependent manner
Source: Nat Commun. 2024 May 27;15:4496. doi: 10.1038/s41467-024-48828-7 (PMC11530556; doi:10.1038/s41467-024-48828-7)
Supplement: Supplementary file 1 — Supplementary information [file 41467_2024_48828_MOESM1_ESM.pdf]

Supplementary Materials for

Structural Domain in the Titin N2B-us Region Binds to FHL2 in a Force-Activation Dependent Manner

Yuze Sun, Xuyao Liu, Wenmao Huang, Shimin Le, and Jie Yan
Corresponding author: phyyj@nus.edu.sg

The PDF file includes:

Supplementary Note 1-3
Figure. S1 to S21
Supplementary Table S1-2
References

Other Supplementary Materials for this manuscript include the following:

Data S1

Figure S1

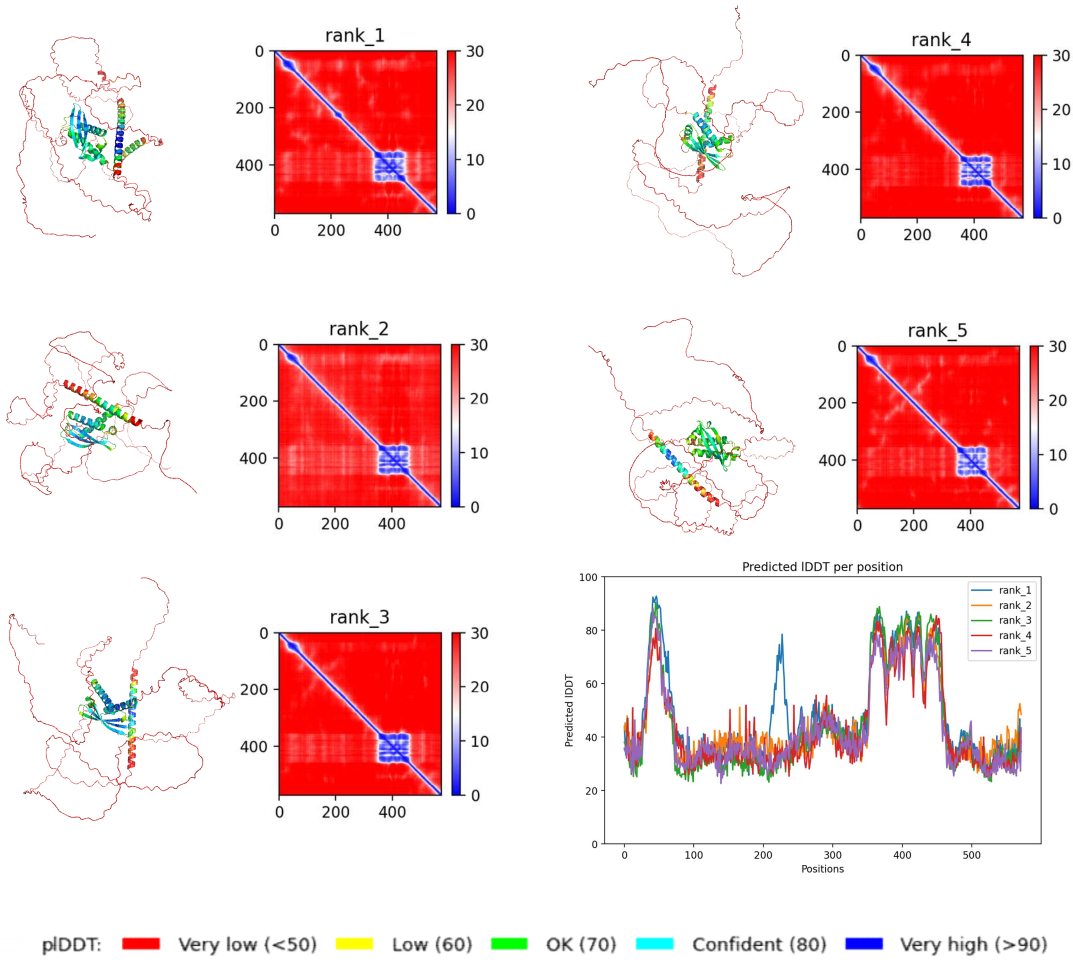

Figure S1 AlphaFold2 predicts the structure of full length N2B-us. ~115a.a. structural domain is predicted in N2B-us in all the five given models, which are colored by pIDDT value. PAE plot on the right of each panel.

Figure S2

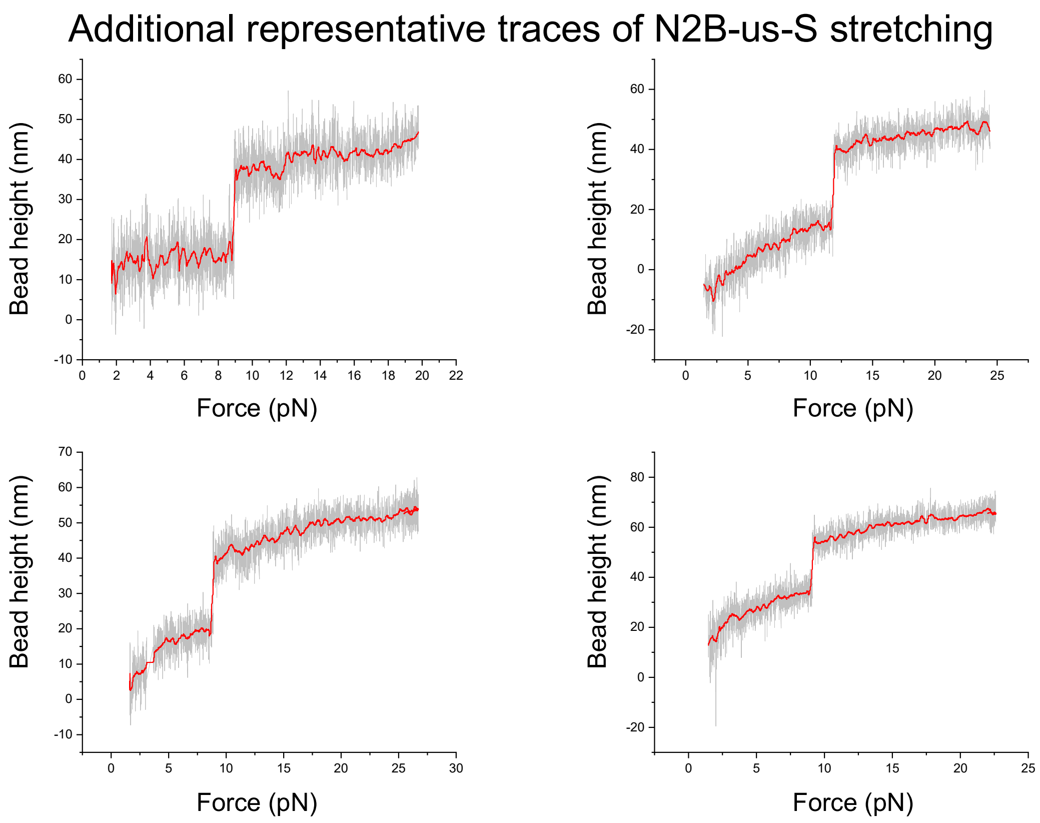

Figure S2 Additional loading force traces of N2B-us-S. All the traces show stepwise bead height change at force around 10pN.

Figure S3

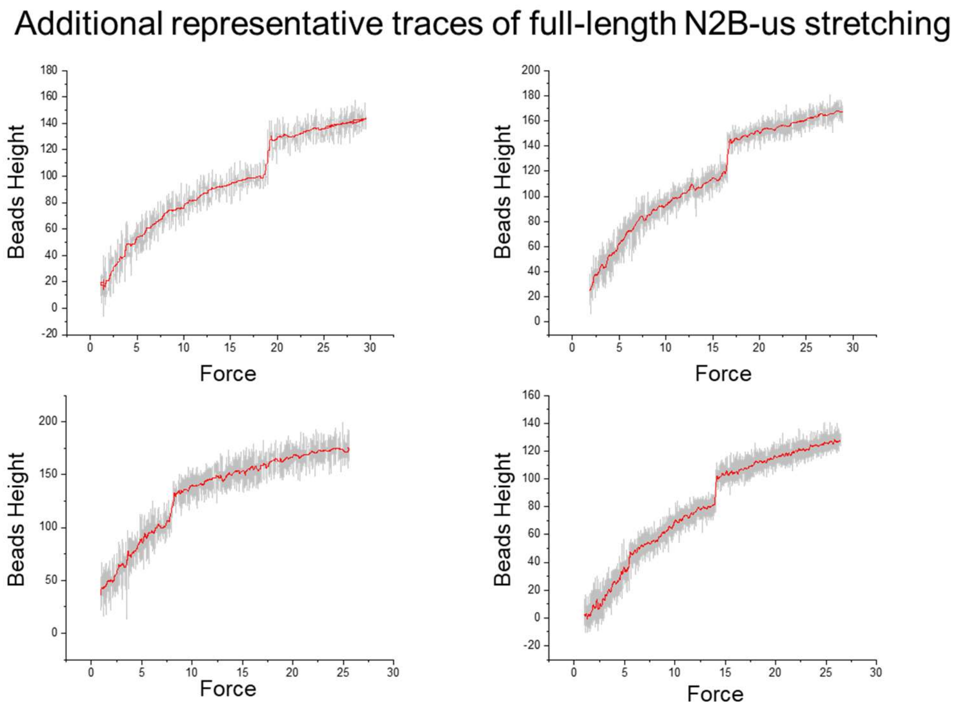

Figure S3 Additional loading force traces of full-length N2B-us. All the traces show stepwise bead height change at force around 10pN.

Figure S4

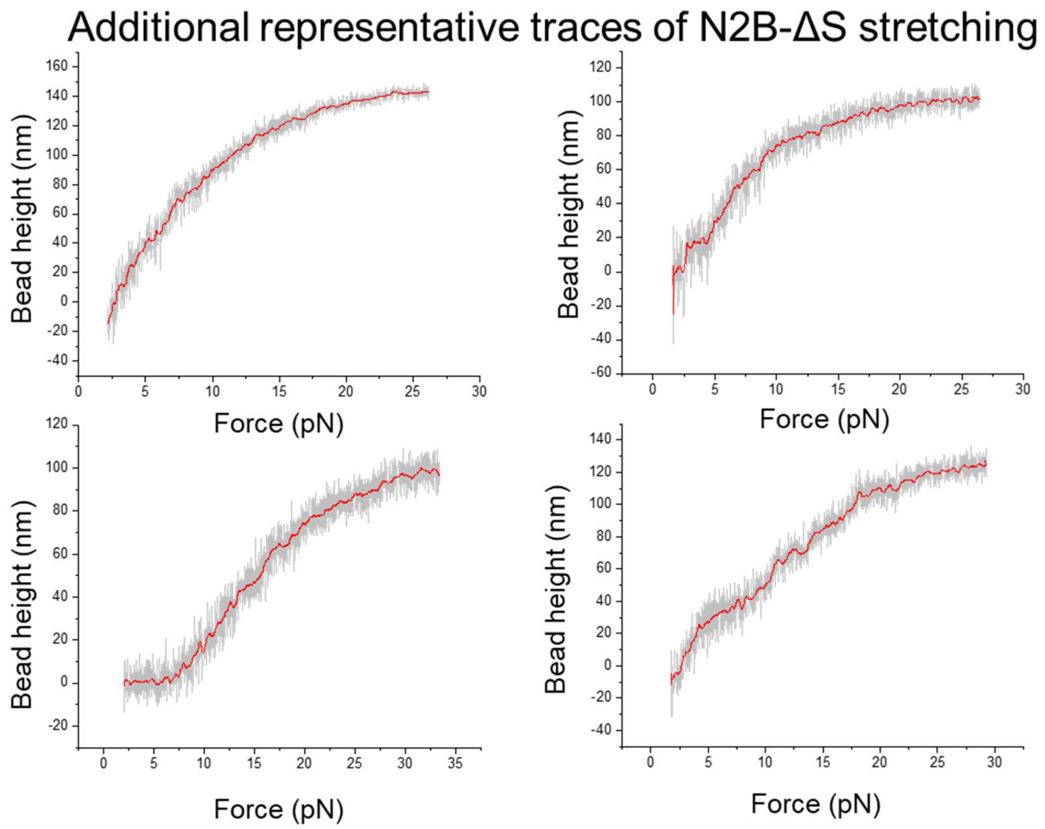

Figure S4 Additional loading force traces of N2B-ΔS. All the traces don't show stepwise bead height change.

Figure S5

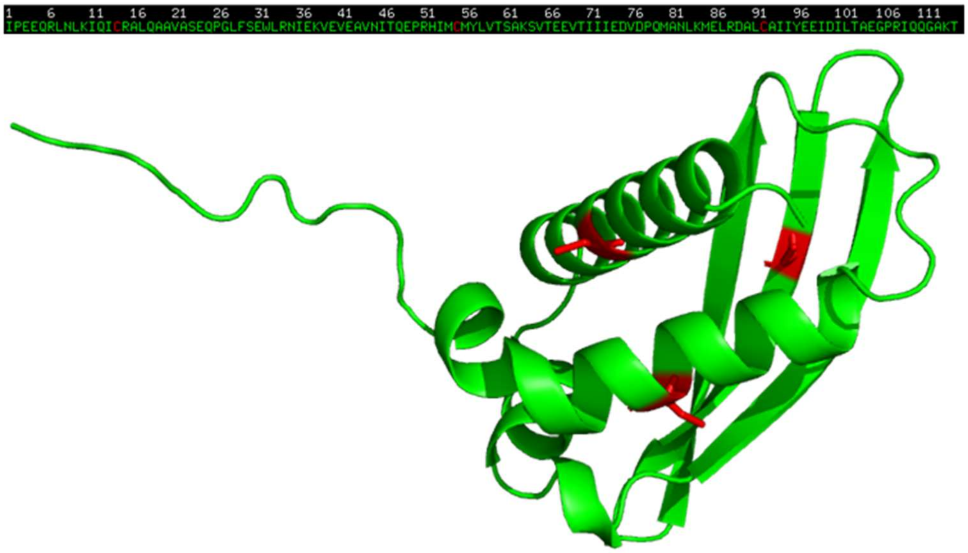

Figure S5 positions of cystines in N2B-us structural domain. In total three cystines in N2B-us structural domain, which are indicated by red color. Their relative position suggests that disulfide bond is not likely form inside the structural domain when the domain is folded.

Figure S6

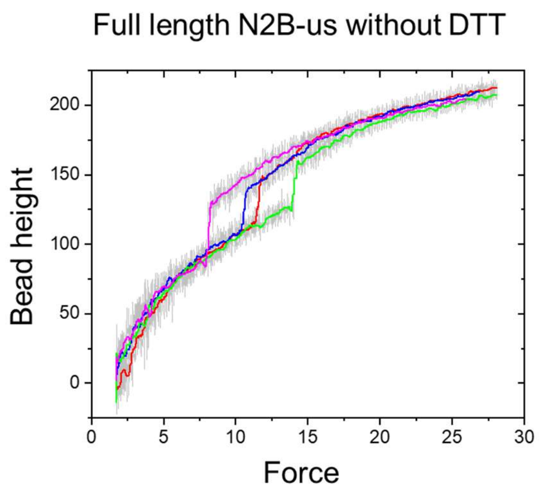

Figure S6. Full length N2B-us without DTT in the buffer. Full length N2B-us stretched by loading force of 1pN/s. gray lines are the raw data, colored lines are smoothed curve indicating four different cycles of stretching. The unfolding step caused by the structural domain still exists.

Figure S7

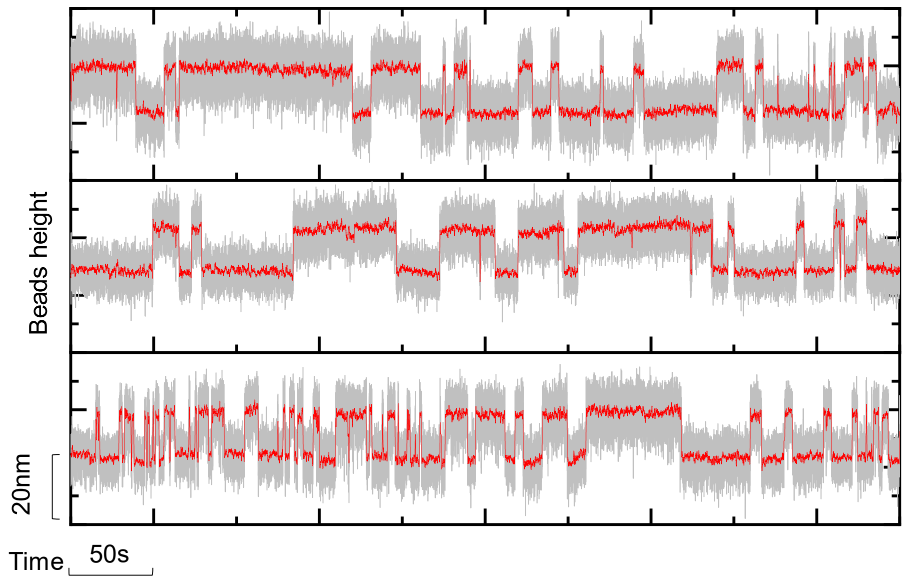

Figure S7 Three tethers of N2B-us-S construct under 5.5pN undergo unfold and refold. Red line is 100 FFT transformation smooth form the original data in grey. Beads heigh goes up and down with ~15nm long step indicate the unfolding and refolding of the structural domain.

Figure S8

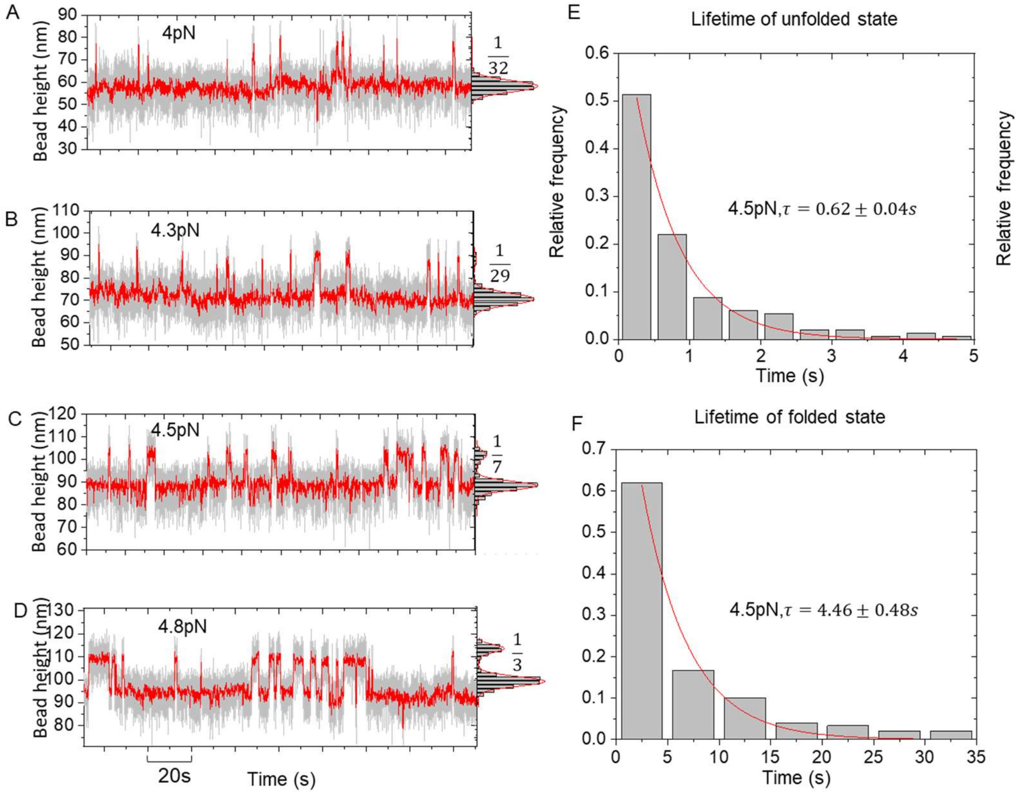

Figure S8 N2B-us structural domain in physiological temperature. (A-D). N2B-us structural domain at physiological relevant temperature (37°C) from 4pN-4.8pN. The bead height undergoes stepwise up-down fluctuations indicating the dynamic unfolding and refolding of the N2B-us structural domain. The panels on the right axis are the distribution histogram of the bead height. When force increases the probability of the unfolded state increases. (E-F). The histograms for the lifetimes of N2B-us structural domain in the unfolded and folded states at 4.5±0.5 pN. The average lifetimes τ can be obtained from fitting the histogram using exponential decay function Ae^(-t/τ).

Figure S9

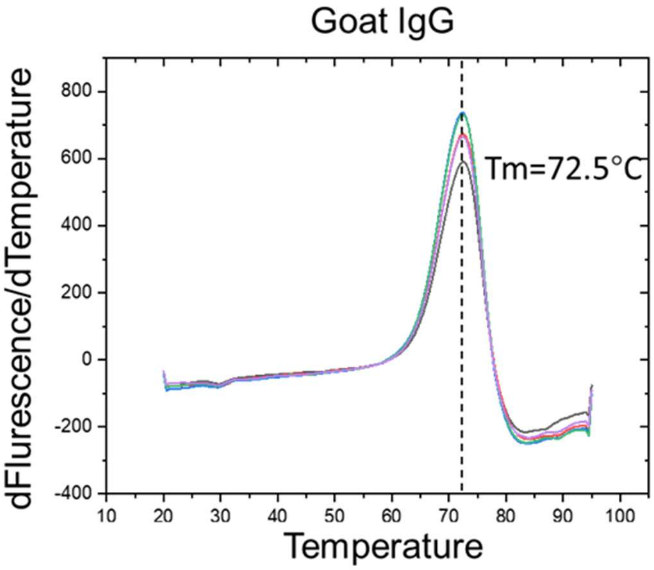

Figure S9 protein thermal shift assay for Goat IgG. Goat IgG protein used for control which has a melting temperature of 72 degrees. Five different color curves indicate experiment in five different independent wells.

Figure S10

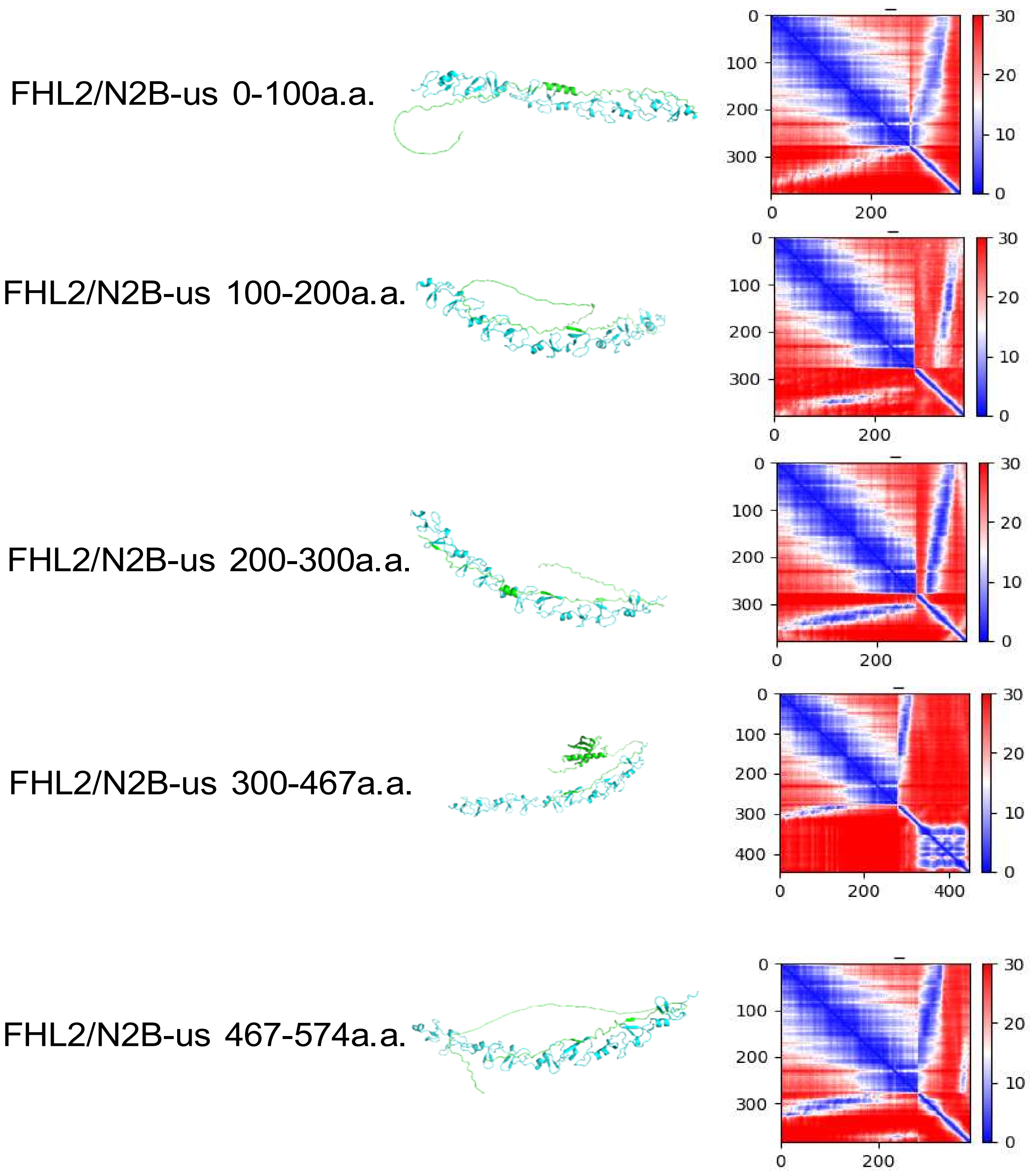

Figure S10. Structure of FHL2/N2B-us fragment complexes predicted by AlphaFold2. Five subsegments of N2B-us in complex with FHL2, revealing FHL2 LIM domains binding sites in each of the segment. The structural domain is retained in the segment of 300-467 a.a. The blue area in the PAE plot on the right indicate the binding interface between FHL2 and N2B-us fragments.

Figure S11

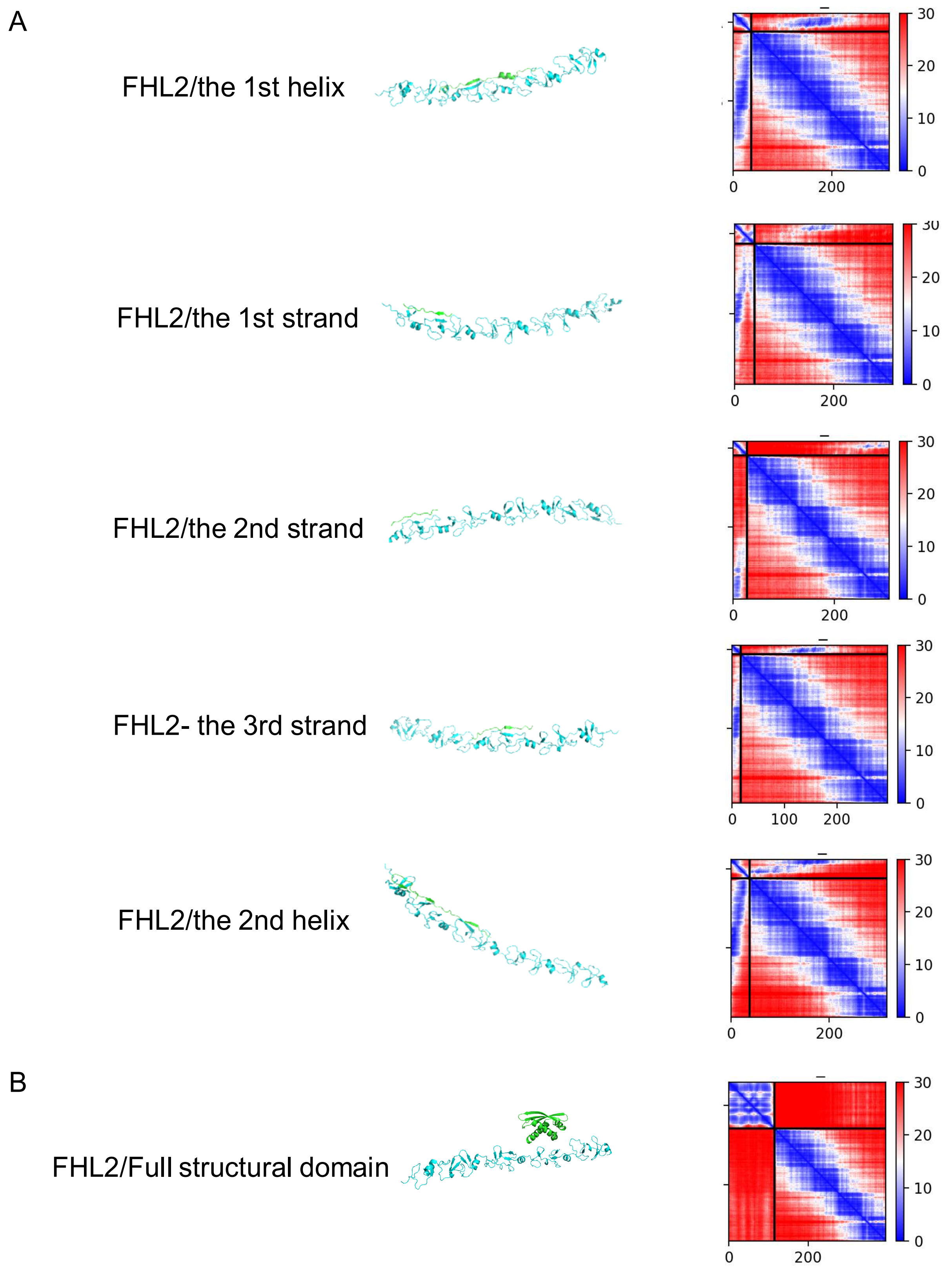

Figure S11. Structure of FHL2/N2B-us structural domain fragments complex predicted by AlphaFold2 (A). The structure of each helical/strand region of N2B-us structural domain in complex with FHL2. On the right is the corresponding PAE plot. They all show confident binding sites. **(B)** The whole 115 a.a. long sequence of N2B-us structural domain in complex with FHL2. The structure and PAE plot show no binding.

Figure S12

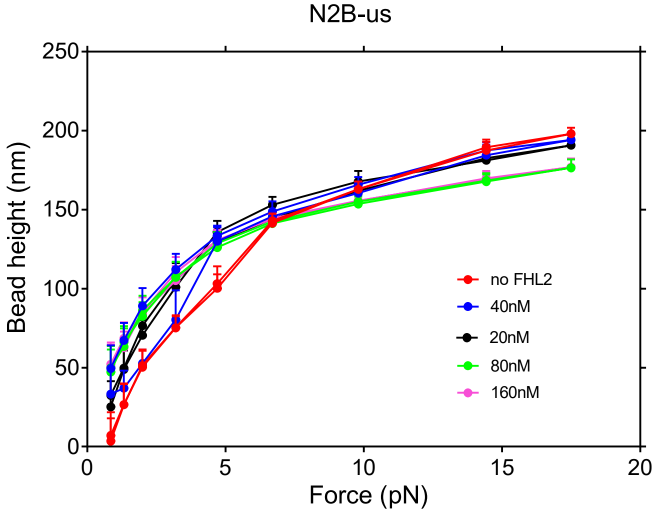

Figure S12 Force-extension curve of N2B-us with different concentration of FHL2. Force-extension curve was made with FHL2 added with increasingly higher concentration. The rigidifying effect of N2B-us was increasing with the concentration of FHL2 increasing. The error bars indicate standard deviation.

Figure S13

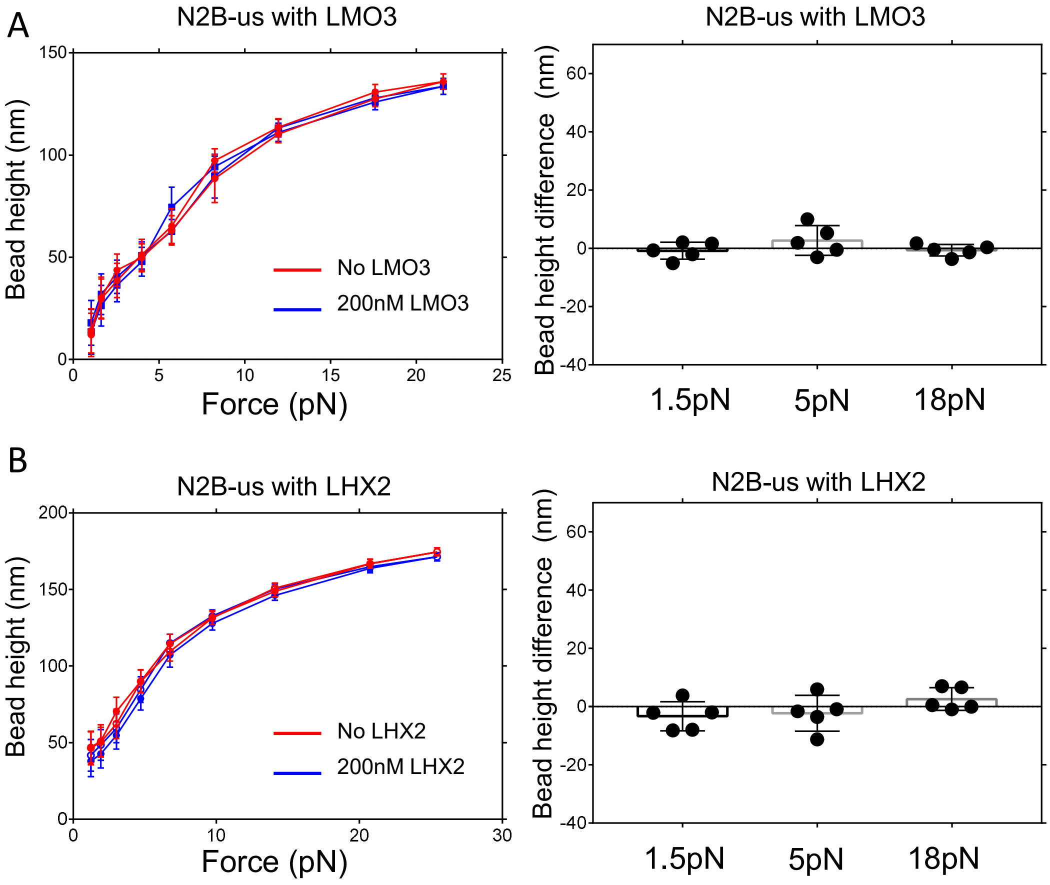

Figure S13 Force-extension curve of N2B-us with LMO3/LHX2. A). Adding of 200nM LMO3 protein to the buffer, N2B-us force-extension curve was not affected. The error bars indicate standard deviation. On the right is the quantification from multiple experiments, bead height differences are around zero. B). Same experiment for LHX2. No binding induced curve shifting observed neither.

Figure S14

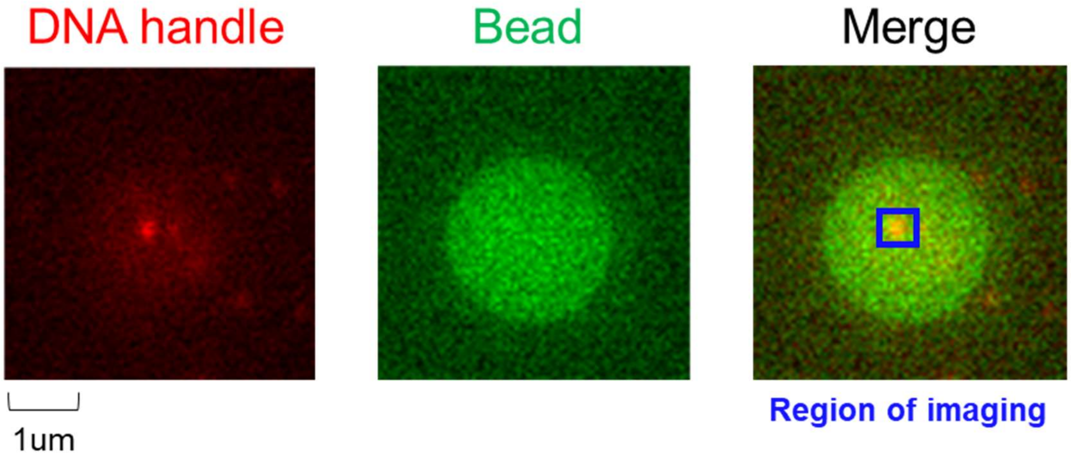

Figure S14 Region selected for TIRF-magnetic imaging experiments. The DNA handle, dyed with Sytox Orange, is visible in the red channel. Tether location is determined by the DNA handle position. The region with area of 576nm*576nm was selected as the region of imaging.

Figure S15

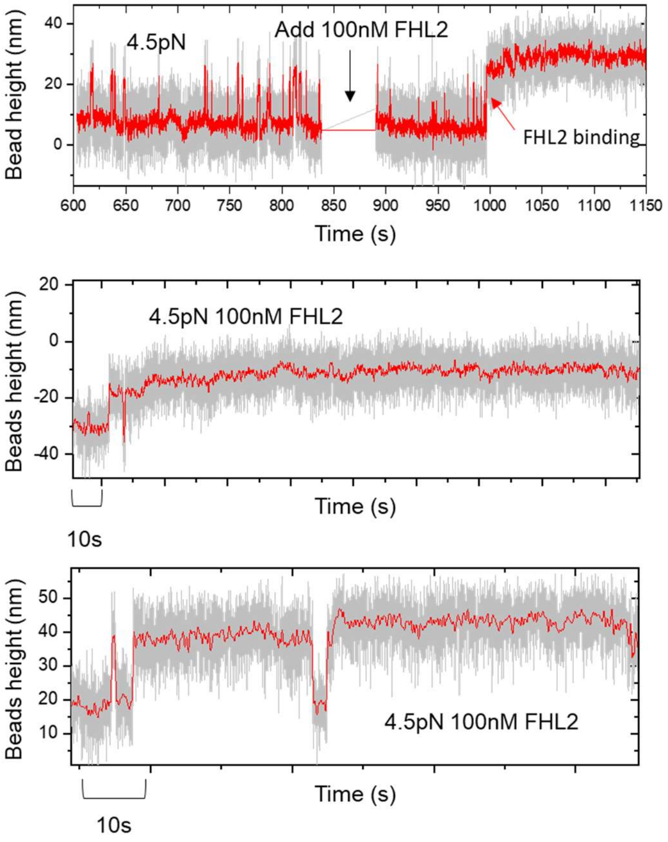

Figure S15 FHL2 binding N2B-us-S at 4.5pN, 37 degree inhibits its refolding. Upper panel: A representative time trace of the bead height of an N2B-us-S tether at 4.5±0.5 pN, 37°C, where dynamic unfolding and refolding are observed. After adding 100nM FHL2 (black arrow), the bead height stably remains at the level corresponding to the unfolded state, indicating binding of FHL2 that blocks the refolding of N2B-us structural domain (red arrow). Lower panel: two other examples.

Figure S16

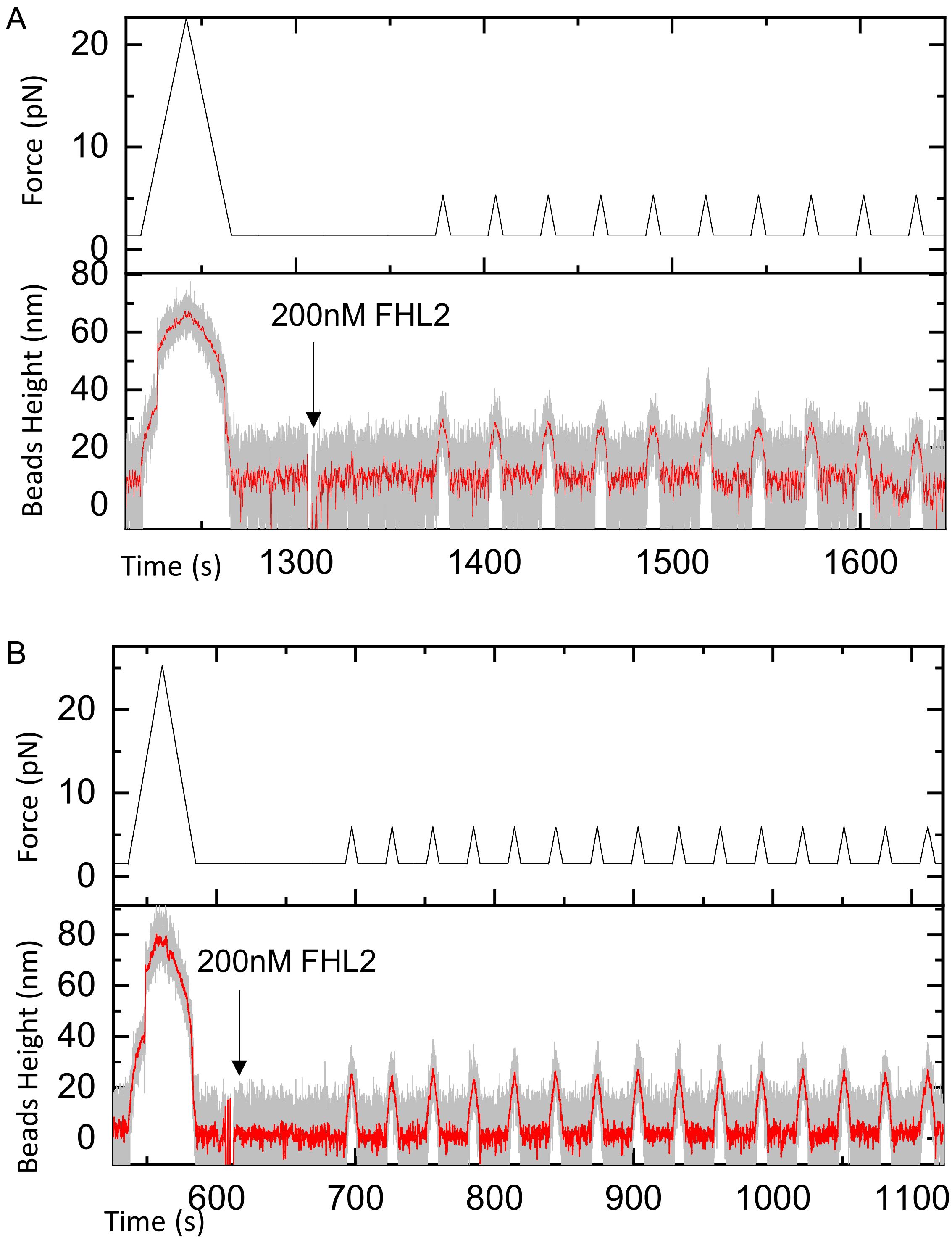

Figure S16 loading force from 1-6pN for N2B-us-S in 200 nM FHL2. (A). After 1 cycle of loading force from 1pN to ~25pN and back to 1pN with loading rates 1pN/s and -1pN/s, respectively, 200 nM FHL2 was add to the buffer as indicated by the arrow. Subsequent loading forces from 1pN to 6pN didn't induce any binding of FHL2 to N2B-us. **(B).** Similar results in another bead.

Figure S17

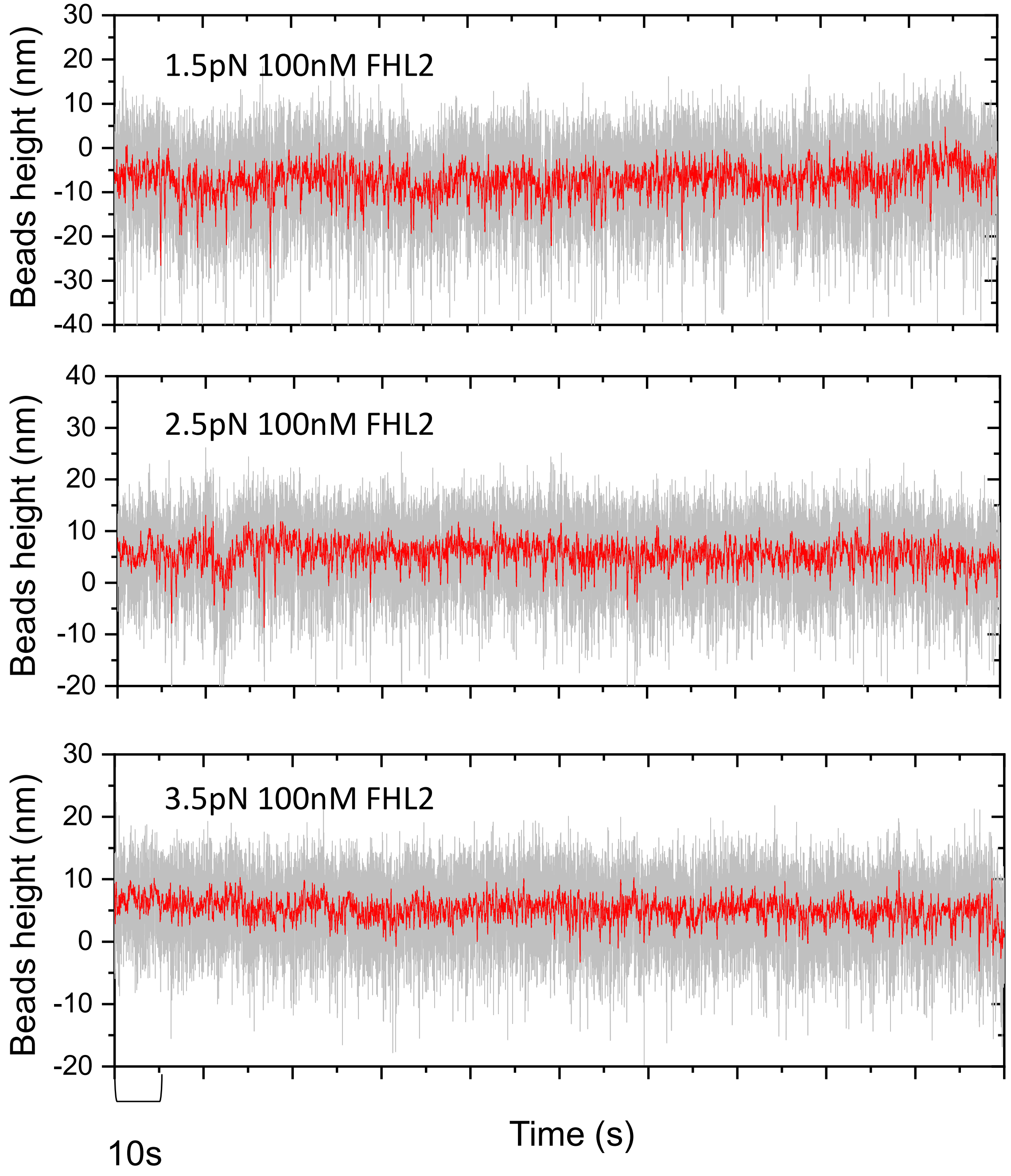

Figure S17 Adding FHL2 to N2B-us-S at 1.5, 2.5, 3.5pN,37 degrees. The bead was applied with forces of 1.5pN, 2.5pN, 3.5pN, respectively. After adding FHL2 to the buffer at 37 degrees, the bead height remains unchanged. Indicating that low force cannot induce FHL2 binding to N2B-us-S.

Figure S18

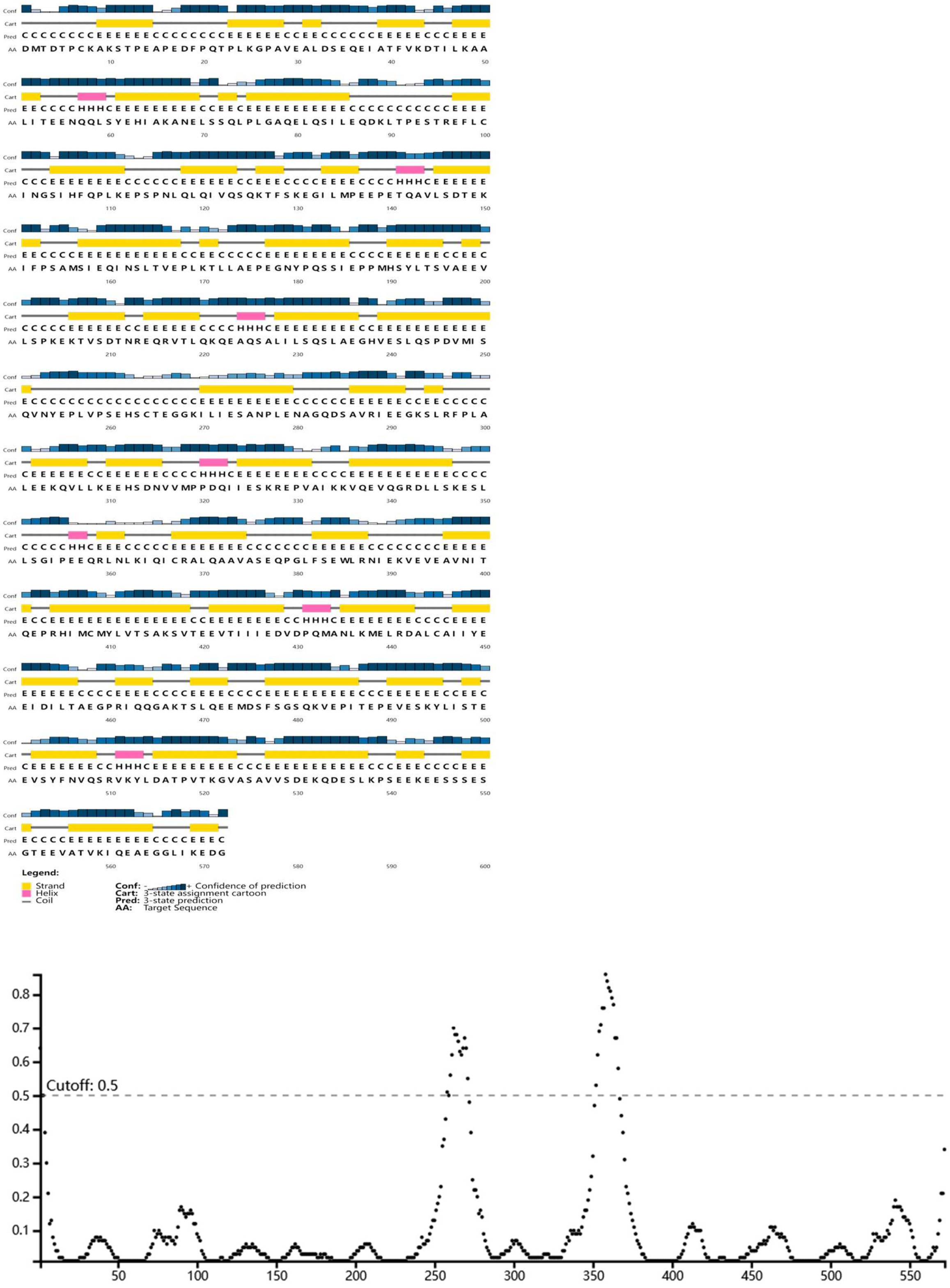

Figure S18 DISOPRED and PSIPRED prediction for N2B-us. The rsults suggest that there are multiple secondary strcutures exist in N2B-us

Figure S19

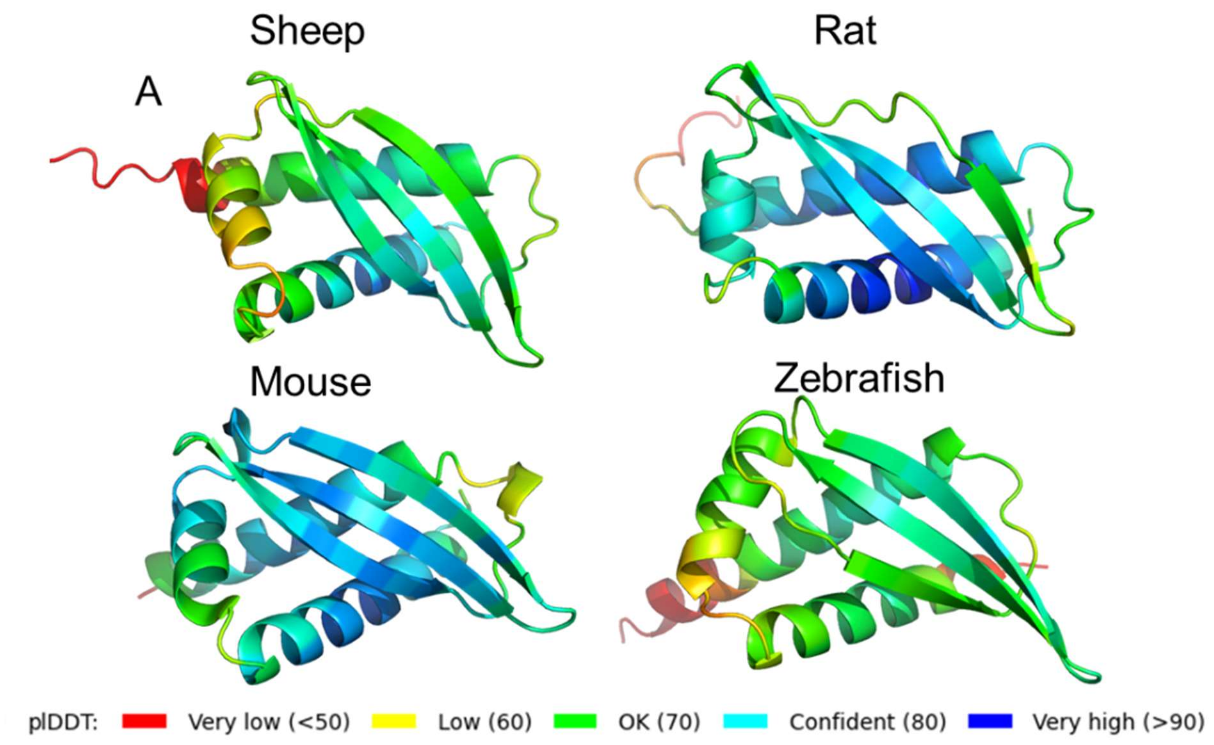

B

```
T-COFFEE, Version_11.00 (Version_11.00)
Cedric Notredame
SCORE=98
*
 BAD AVG GOOD
*
human       :  99
mouse       :  99
zebrafish   :  97
rat         :  99
sheep       :  98
cons        :   9

human       IPEEQRLNLKIQICRALQAAVASEQPGLFSEWLRNIEKVEVEAVNITQEPRHIMCMYLVTSAKSVTEEV
mouse       MPEEQRLHLKTQVRRALQAAVAREQANLFSEWLRNIDKVEVTAVNFTQEPKRILCTYLITSVSSLTEEL
zebrafish   -PKSHSLDIKHQLKSTLATAVALDQPLILADFVESLGVVEIHEVKVRKEPKYLMFTYLITSASA-PLEI
rat         IPKEQRLHLKTQVRRALQAAVAREQASLFSEWLRNIDKVEVTAVNFTQEPKRILCTYLITSVKSLTEEL
sheep       IPEEQRLNLKTQIRSALQAAVASEQLSLFSEWLRNIEQVEVKAVDFTQEPNCIMCTYLITSVKSLTEEL

cons         *:.: *.:* *:  :* :*** :*  :::::..:  **:  *.. :**. ::  **:**..: . *:


human       TIIIEDVDPQMANLKMELRDALCAIIYEEIDILTAEGPRIQQGAKT
mouse       TVTIEDIDPQMANLETGLKDALCSIVCEERNILMAE----------
zebrafish   TIAFEGEYPQTADLRSELQAAFYSIVYREQNVLTSEQP--------
rat         TLTIEDIDPQMANLETELKDALCSIVCEERNILMAEDPRFQEED-K
sheep       TLTIKGIDPQMADLKTELKEALCSI-CEEMNILTAEEPGIEK----

cons        *: ::.  ** *:*.  *: *: :*  .* ::* :*
```

Figure S19 Titin N2B-us structural domain in other organisms. (A). Similar structural domains in mouse, rat, sheep, and zebrafish were predicted by AlphaFold2. Color indicates prediction confidence. (B). sequence alignment of N2B-us structural domain sequences from different organisms. They show high similarity.

Figure S20

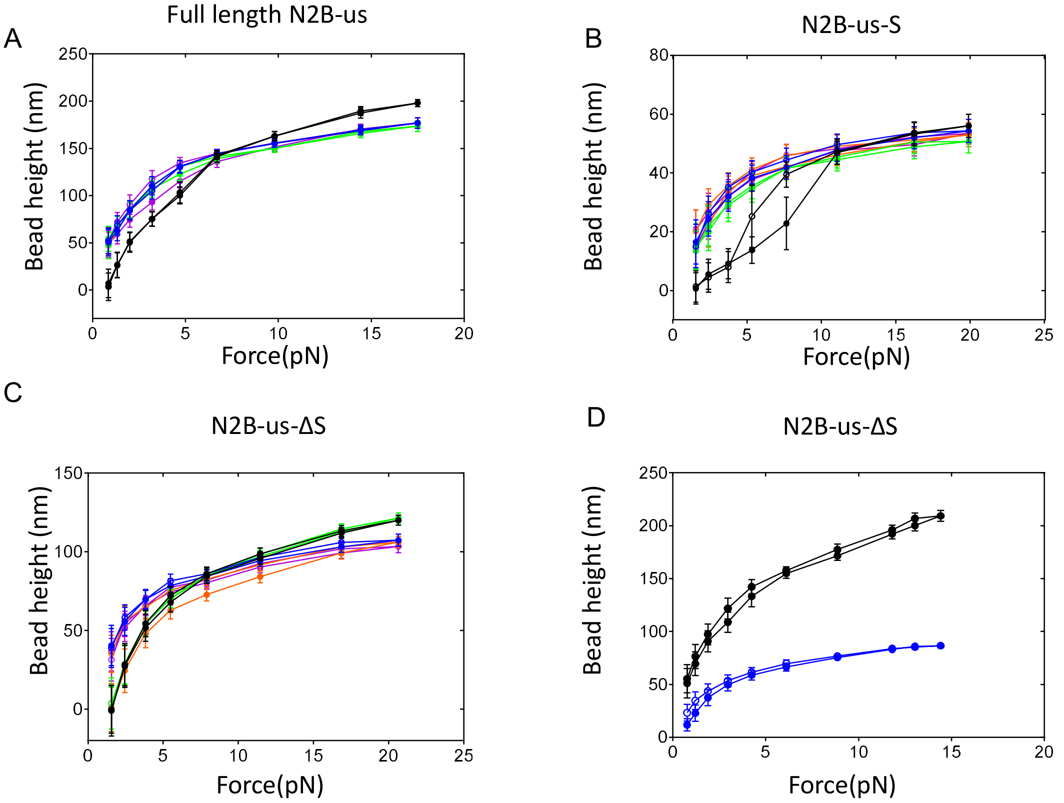

Figure S20 Multiple cycles of force extension curves for N2B-us constructs binding with FHL2. Black curve represents for the bead height before introducing FHL2, colored curves represent for bead height after introducing 200nM FHL2**. (A).** 1 cycle force-extension characterization of full length N2B-us without FHL2 and 4 cycles of force-extension of N2B-us in 200nM FHL2. **(B-C).** same characterization for N2B-us-S and N2B-us-ΔS constructs. **(D).** An extreme case of FHL2 induced looping of N2B-us. In a N2B-us-ΔS construct, after low concentration (10nM) FHL2 introduced and wait for more than 1000s at 1pN force, the height of bead dramatically decreased.

Figure S21

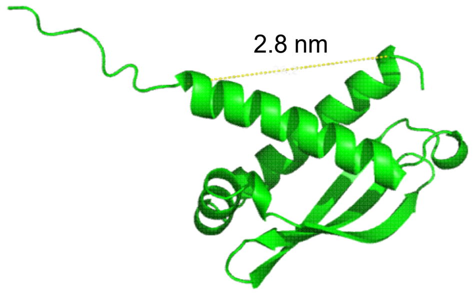

Figure S21 Measurement of N- to C- terminal distance of folded N2B-us structural domain based on the prediction of AlphaFold2. PDB file was obtained from AlphaFold2 prediction based on 115 a.a. long N2B-us structural domain sequence. The measurement is from 356E to 458E, the distance is ~2.8nm.

Supplementary Notes

Note1 Theoretical force-dependent step-size of N2B-us structural domain unfolding/rupturing transitions

A folded domain or complex can be considered as a rigid body. Hence, the force-extension curve of a folded domain or complex is determined by the rigid rotation fluctuation of a rigid body with a characteristic length b ~ 2.8 nm (Figure S21), estimated from the PDB file predicted by AlphaFold2, which is the distance between the two force-attaching points on the stable folded core. The corresponding force-extension curve can be described by the freely jointed chain polymer model with a single segment:

$$x^{FJC}(F) = b\left(coth\left(\frac{Fb}{K_BT}\right) - \frac{K_BT}{Fb}\right) \quad (2),$$

where $K_BT = 4.1\ pN \cdot nm$ at room temperature.

The force-dependent step sizes during unfolding of the domain can be described assuming the unfolded state a randomly disordered polypeptide chain, using the worm-like chain (WLC)[1] polymer model with a reasonable bending persistence length of A ~ 0.8 nm [2]:

$$\frac{FA}{K_BT} = \frac{1}{4\left(1 - \frac{x^{WLC}(F)}{L}\right)^2} - \frac{1}{4} + \frac{x^{WLC}(F)}{L} \quad (3).$$

Here $L = n * 0.38$ nm is the contour length of the unfolded state, where n is the number of residues of the folded core (103 a.a.). The force-dependent unfolding/rupturing step size is the extension differences of the domain between the unfolded and folded states at the transition (unfolding/refolding) force, i.e.,

$$\Delta x(F) = x^{WLC}(F) - x^{FJC}(F) \quad (4).$$

A catachrestic force of $\sim \frac{k_BT}{A}$ is needed to extend a randomly coiled peptide polymer to half of its contour length. Over the typical bending persistence length range, 0.4 nm – 0.8 nm[3-5] of peptide polymer, the half-contour length-extension tensile force is over a range of 5-8 pN.

Note2 Error estimation for folding energy calculation

The folding energy was calculated through the equation:

$$\Delta G_0 = -k_BT\ln\left(\frac{p_{fold}}{p_{unfold}}\right) + \int_0^F (x_0(f) - x_u(f))df \quad (1)$$

The error of folding energy calculation came from two parts: one is the standard error of probability ratio in the first term, recorded as δ_1. Another is the force calibration error in magnetic tweezer experiments, which is ~10%, exists in the second term, recorded as δ_2.

To estimate the value of δ_1, the dwell times of unfold state and refold state were collected and kept in two sets U_{fold} and U_{unfold}, each contains 132 elements. The average unfolding state dwell time was 7.56s, while the average folding state dwell time was 7.86s. A python code-based bootstrap error estimation was performed. In each simulation, 132 elements were selected with replacement from U_{fold} and U_{unfold},

respectively. $G_0 = -k_B T l n\left(\frac{p_{fold}}{p_{unfold}}\right)$ was calculated accordingly, and average value were obtained from the 132 G_0 values. 1000 simulations have been performed and the $\delta_1 = Var(G_0)$ was calculated to be $0.26 k_B T$.
In force calibration, the force has a variance of ~10%. By error propagation calculation, δ_2 was calculated to be $1.9\, k_b T$. Because δ_2 and δ_1 are independent, the total error equals to $\sqrt{\delta_2{}^2 + \delta_1{}^2}$=1.91 $k_b T$

Note3 Estimation of force range on each titin
Human cardiomyocyte I band of titin extends from 50 nm to 300 nm. To estimate the force range acting on each titin molecule within the I band, we utilize two models: for the disordered regions, which is 1019 a.a. long, we used the Worm-Like Chain (WLC) model:

$$\frac{FA}{K_B T} = \frac{1}{4\left(1 - \frac{x^{WLC}(F)}{L}\right)^2} - \frac{1}{4} + \frac{x^{WLC}(F)}{L} \quad (3)$$

In this case, we take the persistence length A=0.8nm, and the length of each amino acid to be 0.38nm. For the 41 Ig-like domains we used the Freely Jointed Chain (FJC) model:

$$x^{FJC}(F) = Nb\left(\coth\left(\frac{Fb}{K_B T}\right) - \frac{K_B T}{Fb}\right) \quad (2)$$

In this case, we take 4.3nm for the length of each folded Ig domain. The total force required to achieve extensions from 50 nm to 300 nm is calculated by integrating the extensions derived from both models, ensuring the total extension matches the specified range. The force that aligns with the total specified extension is solved iteratively using a python code. The calculation shows that the force range on each titin is ~1-5pN.

Supplementary Table S1 Primers used in the study.

N2B structural domain	F	gaaagagctggGAGGCGGTAGCGGaAAGCTTATTCCAGAAGAACAGCGTCTGA
	R	acctcaatcaGTCCTGAACCACCcCCctcgagGGTTTTTGCACCCTGCT
Backbone for N2B structural domain	F	GGTGGTTCAGGACTGATTGAGGTAGAGAAACCACTGTATGGCGTAGA
	R	TTtCCGCTACCGCCTCCCAGCTCTTTCACTTTCAGGTTGGCAGCTG
delete N2B structural domain	F	AGCCTGCAAGAAGAAATGGATAGCTTTAGCGGTA
	R	ACCGCTCAGCAGTGATTCTTTGCTCAGCAG
pGEX-N2B-S	F	AAAACCTAAGAATTCCCGGGTCGACTCGAGCGGCCG
	R	TGGAATGGATCCACGCGGAACCAGATCCGATTTTGG
DNA Handel	F	ATGAGATAAGCAGCGCAACACCCTT
	R	GTGCCGTGGAGTAGCTCATCTGG

Supplementary Table S2 DNA Fragment used in the study.

Pgex-N2B-us-S	ATCTGGTTCCGCGTGGATCCGGCCTGAACGATATTTTTGAAGCCC<br>AGAAAATTGAATGGCATGAAATTCCAGAAGAACAGCGTCTGAAT<br>CTGAAAATTCAGATCTGTCGTGCACTGCAGGCAGCAGTTGCAAGC<br>GAACAGCCTGGTCTGTTTAGCGAATGGCTGCGTAATATTGAAAAA<br>GTTGAAGTGGAAGCCGTGAACATTACCCAAGAACCGCGTCATAT<br>CATGTGTATGTATCTGGTTACCAGCGCAAAAAGCGTGACCGAAG<br>AAGTTACCATTATCATCGAAGATGTGGATCCGCAGATGGCAAAC<br>CTGAAAATGGAACTGCGTGATGCACTGTGTGCAATCATTTATGAA<br>GAAATTGACATCCTGACCGCAGAAGGTCCGCGTATTCAGCAGGG<br>TGCAAAAACCGCACATATTGTTATGGTTGATGCATACAAACCGAC<br>CAAATAAGAATTCCCGGGTCGACTCGA

1 Marko, J. F. & Siggia, E. D. Stretching dna. *Macromolecules* **28**, 8759-8770 (1995).

2 Winardhi, R. S., Tang, Q., Chen, J., Yao, M. & Yan, J. Probing Small Molecule Binding to Unfolded Polyprotein Based on its Elasticity and Refolding. *Biophys J* **111**, 2349-2357, doi:10.1016/j.bpj.2016.10.031 (2016).

3 Bouchiat, C. *et al.* Estimating the persistence length of a worm-like chain molecule from force-extension measurements. *Biophys J* **76**, 409-413 (1999).

4 Hugel, T. *et al.* Elasticity of single polyelectrolyte chains and their desorption from solid supports studied by AFM based single molecule force spectroscopy. *Macromolecules* **34**, 1039-1047 (2001).

5 Rief, M., Gautel, M., Oesterhelt, F., Fernandez, J. M. & Gaub, H. E. Reversible unfolding of individual titin immunoglobulin domains by AFM. *science* **276**, 1109-1112 (1997).
